# Effects of flavonoids on atherosclerosis in ApoE^−/−^ mice: a systematic review and meta-analysis

**DOI:** 10.3389/fphar.2026.1816659

**Published:** 2026-05-29

**Authors:** Peiqi Gao, Man Gong, Junchao Wu, Songru Guo, Shuni He, Chengfu Su

**Affiliations:** 1 Henan University of Chinese Medicine, Zhengzhou, China; 2 The Second Clinical Medical College of Henan University of Chinese Medicine, Zhengzhou, China; 3 Collaborative Innovation Center of Research and Development on the Whole Industry Chain of Yu-Yao, Henan Province, Henan University of Chinese Medicine, Zhengzhou, China

**Keywords:** anti-inflammatory, antioxidant, ApoE^−/−^ mice, atherosclerosis, flavonoids, meta-analysis

## Abstract

**Background:**

Atherosclerosis (AS) plays a key role in cardiovascular diseases (CVDs) through dysregulated lipid metabolism. Flavonoids have shown promise in combatting AS due to their broad biological impacts. However, a comprehensive assessment of the methodological quality and outcomes of these studies is still lacking. This study con-ducted a rigorous systematic review and meta-analysis of research on the therapeutic potential of flavonoids in ApoE^−/−^ mice, a recognized atherosclerosis model.

**Methods:**

Relevant literature was sourced from databases like PubMed, Web of Science et al. The studies were assessed for bias using the Cochrane Reviewers Handbook 6.1.0 tool. Data were ana-lyzed for heterogeneity, subgroup analysis, sensitivity analysis, and publication bias using Review Manager 5.4. The meta-analysis synthesized lipid data using standardized mean differences, employed fixed or random effects models based on heterogeneity (*I* ^*2*^ statistic), and conducted sensitivity and publication bias analyses.

**Results:**

A systematic search of four databases identified 11 studies meeting the inclusion criteria, all investigating flavonoids in male ApoE^−/−^ mice. A meta-analysis of the extracted data, using random-effects models due to high heterogeneity (*I* ^*2*^ ≥ 75%), found that flavonoid administration significantly reduced blood levels of LDL-C, TC, and TG while increasing HDL-C. Sensitivity analysis confirmed the robustness of these results, though funnel plots and statistical tests indicated potential publication bias for three of the four lipid markers. All 11 studies also reported a reduction in aortic plaque area, with proposed mechanisms primarily involving antioxidant effects and the inhibition of inflammatory pathways.

**Conclusion:**

This study synthesizes pre-clinical evidence indicating that flavonoids are promising therapeutic agents for atherosclerosis, primarily through lipid modulation, plaque reduction, and pleiotropic antioxidant/anti-inflammatory mechanisms. The high heterogeneity and potential publication bias highlight the need for more standardized, high-quality animal studies to confirm these effects and better define optimal treatment protocols before clinical translation.

## 1 Introduction

Atherosclerosis (AS) is a pathological condition resulting from dysregulated lipid metabolism (Melendez et al., 2017; Borén et al., 2022), implicated in the onset of multiple cardiovascular conditions including coronary heart disease and peripheral vascular disease. As per the findings of the 2023 World Heart Report published by the World Heart Federation (WHF), cardiovascular disease has consistently been the leading cause of death globally for an extended period. In 2021, an estimated 20.5 million deaths are projected to occur globally due to cardiovascular disease, representing approximately one-third of all recorded deaths. Among these fatalities, 3.8 million are associated with high levels of low-density lipoprotein cholesterol (LDL-C) (H). The pathophysiological mechanisms of atherogenesis are complex, and evidence from various research studies supports the significant role of LDL-C in the progression of atherosclerotic cardiovascular disease (ASCVD) (Ference et al., 2017). Recently, statins (3-hydroxy-3-methylglutaryl CoA reductase inhibitors) have emerged as the preferred medications for preventing and managing atherosclerosis by lowering LDL-C levels (Bruikman et al., 2017; Fan and Watanabe, 2022). However, their clinical utility has been limited due to certain toxic side effects (Stroes et al., 2015; Ward et al., 2019). Hence, it is imperative to explore novel pharmaceutical interventions for the management of atherosclerosis.

Flavonoids, which are polyphenolic compounds commonly present in various plant sources (Di Lorenzo et al., 2021), demonstrate a variety of advantageous characteristics, such as antiviral, anticancer, anti-inflammatory, and antioxidant effects (Durazzo et al., 2019; Ullah et al., 2020; Badshah et al., 2021). Recent research has demonstrated a notable inverse relationship between flavonoid consumption and cardiovascular risk (Maron, 2004; Li et al., 2022). Epidemiological research indicates a positive correlation between the intake of foods high in flavonoids and improved cardiovascular health outcomes (Rana et al., 2022). Flavonoids have been found to potentially confer vasoprotective benefits through mechanisms such as LDL oxidation inhibition, thrombosis reduction, endothelial function improvement, and inflammation reduction (Khan et al., 2021; Ebrahimi et al., 2023; Li et al., 2023).

Apolipoprotein E (ApoE) is an essential component in lipoprotein metabolism, particularly in the elimination of chylomicrons and very low density lipoproteins (Koopal et al., 2016). Insufficiency of ApoE can lead to disruptions in cholesterol balance and the onset of atherosclerosis. ApoE^−/−^ mice serve as commonly utilized animal models in experimental atherosclerosis research due to their rapid reproduction, facile genetic modification, and susceptibility to atherosclerosis (Emini Veseli et al., 2017). Studies have shown that flavonoids present in plants or pharmaceuticals have the potential to prevent and manage atherosclerosis in ApoE^−/−^ mice (Jia et al., 2019). The majority of distinct classes of flavonoids have demonstrated the ability to decrease the lesion area associated with atherosclerosis, yet the specific mechanisms underlying their effects on atherosclerosis vary (Li et al., 2020). However, the effectiveness of flavonoids in mitigating atherosclerosis in ApoE^−/−^ mice is still unclear, with a lack of robust data. Therefore, it is imperative to conduct evidence-based meta-analyses and systematic reviews to further investigate this topic. This study aimed to systematically review and meta-analyze the current body of research to evaluate the efficacy of flavonoids in the treatment of atherosclerosis in ApoE^−/−^ mouse models. Various parameters, including aortic atherosclerotic lesion area, LDL-C, TC, TG, HDL-C, and other serum or plasma markers in control and experimental groups, were utilized as benchmarks for the meta-analysis and systematic review. Additionally, the study sought to further explore the impact of flavonoids on the distribution, plaque formation, and mechanism of atherosclerotic lipids in ApoE^−/−^ mouse models.

## 2 Methods

### 2.1 Search strategies

A comprehensive literature review was conducted to evaluate the impact of flavonoids on arterial plaque and lipid markers in atherosclerosis models. The literature search encompassed major databases including PubMed, Web of Science, EMBASE, and the Cochrane Library, covering publications up to February 2023., utilizing specific search strategies that incorporated keywords, subject headings, and free words as detailed in Supplementary Table S1.

Five researchers (Peiqi Gao, Man Gong, Junchao Wu, Songru Guo, Shuni He) conducted a thorough review of titles and abstracts to identify potential studies. Duplicate studies were eliminated after careful examination of each article’s title and abstract. The reviewers (Gong Man, Chengfu Su) evaluated the abstracts of the publications through screening and independently assessed the eligibility of animal studies based on predetermined criteria. Additionally, relevant data was gathered by manually searching the references of the included studies. Specific search strategies for each database can be found in Supplementary Table S2.

### 2.2 Eligibility criteria

#### 2.2.1 Inclusion criteria

1. Study subjects included ApoE^−/−^ mice of varying ages and genders.
2. Interventions involved administering any dose of flavonoid monotherapy to the experimental group, while the model control group received an equivalent dose of non-functional substances or no treatment.
3. The study assessed outcome measures such as serum or plasma levels of LDL-C, TC, TG, and HDL-C in both control and experimental groups. Additionally, it examined the variation in the area of aortic atherosclerotic lesions between the groups, as well as the underlying mechanisms by which flavonoids influence atherosclerosis.

#### 2.2.2 Exclusion criteria

1. Study subjects: small animals other than ApoE^−/−^ mice were excluded.
2. Interventions: non-flavonoid monotherapy, mainly flavonoid combined with other drugs.
3. Study results: no pre-specified outcome measures or available data.
4. Research forms: *in vitro* experiment, clinical study, cross-over study.
5. Literature type: unrelated to the subject of this study, literature with repeated research content, literature with repeated publication, literature with incomplete data, reviews, reports, systematic reviews, abstracts, case reports and analyses, review articles or patents.

### 2.3 Data extraction

The detailed data from all the studies was extracted by the five researchers, Peiqi Gao, Man Gong, Junchao Wu, Songru Guo, Shuni He individually. Following the completion of information extraction, a process of cross-verification was conducted. In cases where discrepancies arose, reviewers Chengfu Su, Man Gong, engaged in discussion and made judgments. The extracted detailed information is presented below.

1. The essential research details encompass the author’s name and the year of publication.
2. The characteristics of the animal model encompass sex, age, and diet.
3. The study design stipulates the precise dosage and duration of administration for both the treatment group and the control group.
4. The primary outcome measures include assessing serum or plasma LDL-C levels in both the control and experimental groups, along with evaluating alterations in the area of aortic atherosclerotic lesions for each group. Secondary outcome measures encompass the levels of TC, TG, and HDL-C in serum or plasma.
5. The mechanism of action was investigated in each study with regard to the impact of flavonoids on atherosclerosis.

In cases where data were presented in image form, hindering direct access to experimental results, original data were extracted using digital image analysis software (GetData Graph Digi0.20) and recorded to two decimal places.

### 2.4 Risk assessment of literature bias

Six researchers, Peiqi Gao, Man Gong, Junchao Wu, Songru Guo, Shuni He and Chengfu Su employed the Cochrane Reviewers Handbook 6.1.0 bias risk assessment tool for a systematic review (Higgins et al., 2011), incorporating SYCLE’s animal experiments Bias Risk tool (Hooijmans et al., 2014). This review included.

1. Assessment of Random Sequence Generation (Selection Bias): This involves reviewing the methods employed to generate a random sequence for participant allocation.
2. Examination of Allocation Concealment (Selection Bias): This pertains to the evaluation of the methods used to conceal the allocation sequence, ensuring that the assignment of participants to intervention groups cannot be predicted or manipulated.
3. Evaluation of Blinding of Participants and Personnel (Performance Bias): This process assesses the effectiveness of blinding strategies for both participants and investigators during the intervention phase, to prevent performance bias.
4. Assessment of Blinding of Outcome Evaluation (Detection Bias): This involves appraising the blinding methods applied to those measuring the outcomes, to prevent detection bias in the evaluation of results.

Incomplete outcome data (attrition bias): evaluation of whether the data of outcome indicators are completely reported, including the number of lost follow-up and withdrawal, reasons and processing.

5. Selective reporting (reporting bias): evaluation of whether various outcome indicators are reported completely and whether selective reporting is performed.

And

6. Other bias: other bias.

For the evaluation results of the included literature, “Low risk” was used to indicate low risk bias, “High risk” to indicate high risk bias, and “Unclear risk” to indicate uncertain risk bias. In cases of disagreement, the differences in quality assessment will be resolved through discussion with the reviewer (Gong Man).

### 2.5 Data synthesis and meta-analysis

Out of the 11 studies analyzed, three studies (Wu et al., 2018; Wang et al., 2021; Zeng et al., 2021) featured two experimental groups with low dose and high dose, and the high dose was selected for data analysis. The conversion of lipid units was conducted using the MedSci medicine tool (https://m.medsci.cn/scale/show.do?id=6e3d228276) for different data units. The evaluation of atherosclerotic lesion size in aortic tissue and the pharmacological mechanisms affecting atherosclerosis were only briefly summarized and discussed.

The statistical analysis of the collected TC, TG, LDL-C, and HDL-C data was performed by Review Manager 5.4 and Stata 17.0 software. Since all selected indicators were continuous variables, the effect size was determined using the Standardized Mean Difference (SMD), and the 95% Confidence Interval (CI) was employed to assess the effect size. Heterogeneity was assessed through the Q statistic test and I^2^ statistic test values in the forest plot generated by Review Manager 5.4 software. In the Q test, variance component (*Tau* ^*2*^), chi-square value (*Chi* ^*2*^), degree of freedom (*df* = number of studies −1) and p-value are included, but the heterogeneity is mainly determined by the size of P-value. When *P* > 0.1, the included studies were considered to be homogenous, and when *P* ≤ 0.1, the included studies were considered to be heterogeneous. In the *I* ^*2*^ test, the greater the *I* ^*2*^ statistic, the greater the heterogeneity. When *I* ^*2*^ is less than 25%, the level of heterogeneity among the included studies is deemed to be low. When 25% ≤ *I* ^*2*^ ≤ 75%, the heterogeneity among the included studies was moderate. When *I* ^*2*^ is ≥75%, the degree of heterogeneity among the included studies is deemed to be high. Generally, Fixed-effects models (FE) were used for data analysis when the heterogeneity among studies was not obvious (*P* > 0.1 and *I* ^*2*^ < 50%). When the heterogeneity among studies was substantial (i.e., *P* ≤ 0.1 or *I* ^*2*^ ≥ 50%), Random-effects models (RE) were used for data analysis.

Review Manager 5.4 software was employed to create a forest plot for the heterogeneity test. In cases where heterogeneity was present, subgroup analysis was conducted to identify its source. Sensitivity analysis, involving the deletion of individual studies one by one, was performed using Stata 17.0 software to assess changes in heterogeneity. Both Review Manager 5.4 and Stata 17.0 software were utilized for publication bias analysis, including the examination of funnel plots, Begg’s test, and Egger’s test to evaluate the symmetry of the results. The P-values from Begg’s test and Egger’s test were considered.

## 3 Result

### 3.1 Literature search

**FIGURE 1:**
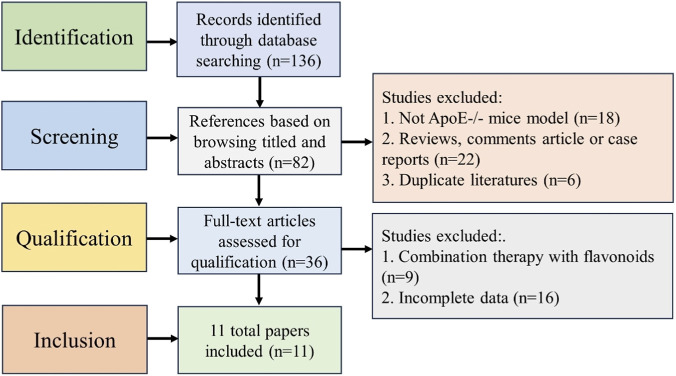
Flow chart of literature retrieval and screening process.

The researchers (Chengfu Su, Ruochen Wang, Bin Wang, Songru Guo, Junchao Wu, and Yinchi Chen) searched four databases, including PubMed, EMBASE, Web of Science, and Cochrane, according to the search strategy, and screened the literature related to the treatment of atherosclerosis in ApoE^−/−^ mice by flavonoids published prior to February 2023 from the establishment of the database. A total of 1,019 records were initially retrieved from all sources, including 136 records identified through database searching. Subsequently, 36 related literature were further screened according to inclusion criteria, 25 were further screened according to exclusion criteria, Ultimately, 11 literature sources meeting the inclusion criteria were included in the final analysis (Wang et al., 2012b; Zeng et al., 2021; Auclair et al., 2009; Wu et al., 2018; Sun et al., 2013; Luo et al., 2020; Qi et al., 2020; Ding et al., 2019; Wang et al., 2018; Wang et al., 2012b; Wang et al., 2021). Refer to Figure 1 for details regarding the screening process.

### 3.2 Data extraction

**FIGURE 2:**
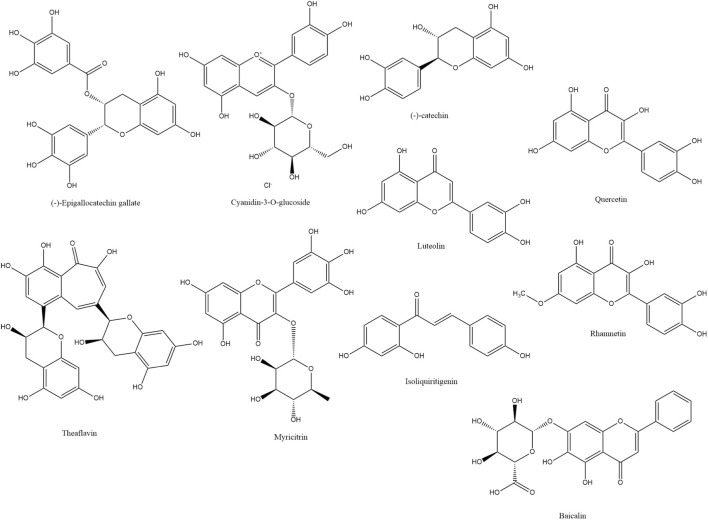
Chemical structure of the drug included in the study.

A total of 11 literature meeting the inclusion criteria were identified, all in English, including 11 independent experimental studies (Table 1). The animal models used in the study were all male ApoE^−/−^ mice. The age of the mice in the studies ranged from five to 10 weeks, with only one study failing to mention the age of the mice (Wang et al., 2021). Anthocyanins, flavonols, chalcones, catechins, and flavones were the focus of investigation in the 11 selected studies (Table 2; Figure 2). In terms of route of administration, eight studies administered the drug by mixing it with the diet, and three studies employed oral gavage. In terms of the source and purity of the drug, nine studies indicated that the drug was purchased, one study isolated the drug experimentally from plants (Sun et al., 2013), six studies mentioned the purity of the drug, four studies did not provide information on the drug’s purity, and only one study did not mention the source and purity of the drug (Wang et al., 2021). In terms of histopathological changes, changes in aortic atherosclerotic plaque area were selected as indicators in all 11 studies. The lesion area was determined by oil red O staining in seven studies, hematoxylin-eosin staining in two studies (Wu et al., 2018; Qi et al., 2020), and the lesion area was determined by oil red O staining and hematoxylin-eosin staining study (Wang et al., 2021). In 1 study, hematoxylin-eosin and masson staining were used to determine the lesion area (Zeng et al., 2021). With respect to serum or plasma markers, 11 studies reported TC markers; TG was reported in nine studies and not reported studies (Luo et al., 2020; Wang et al., 2021). LDL-C was reported in nine studies and not reported studies (Auclair et al., 2009; Wang et al., 2012b). HDL-C was reported in nine studies and not reported studies (Auclair et al., 2009; Luo et al., 2020). The mechanism of action of drugs on atherosclerosis was reported in all 11 studies.

**TABLE 1:** Basic characteristics of the included literatures.

First author and year	Animal model features					Interventions						Outcome indicators
	Species	Age	Gender	Quantity/only	Groups	Quantity/only	Medication	Drug purity	Route of administration	Dose of administration	Duration	
Wang et al. (2012b)	ApoE^−/−^	5 weeks	Male	26	Control group	13	AIN-93G	98% or higher	Feeding	100 mg/kg/d	12 weeks	①②③④
					Experimental group	13	Cy-3-G + AIN-93G		Feeding	100 mg/kg/d	12 weeks	
Zeng et al. (2021)	ApoE^−/−^	6 weeks	Male	30	Control group	10	HFD	98% or higher	Feeding	—	12 weeks	①②③ ④⑤⑥
					Experimental group	10	HFD + TF (low)		Feeding	5 mg/kg	12 weeks	
						10	HFD + TF (high)		Feeding	10 mg/kg	12 weeks	
Auclair et al. (2009)	ApoE^−/−^	10 weeks	Male	20	Control group	10	AIN-93G	—	Feeding	0.02%	6 weeks	①②③⑥
					Experimental group	10	(+)-Catechin + AIN-93G		Feeding	0.02%	6 weeks	
Wu et al. (2018)	ApoE^−/−^ Mice	8 weeks	Male	15	Control group	5	HFD	98%	feeding	—	12 weeks	①②③ ④⑤⑥
					Experimental group	5	HFD + Bai(low)		Feeding	50 mg/kg/d	12 weeks	
						5	HFD + Bai(high)		Feeding	100 mg/kg/d	12 weeks	
Sun et al. (2013)	ApoE^−/−^	6 weeks	Male	20	Control group	10	HFD + CMC-Na	99%	Feeding	-	6 weeks	①②③ ④⑤⑥
					Experimental group	10	HFD + Myricitrin+0.5%CMC-Na		Feeding	50 mg/kg/d	6 weeks	
Luo et al. (2020)	ApoE^−/−^	6 weeks	Male	—	Control group	10 10	HFD	95% or higher	feeding	—	8 weeks	①③④⑥
					Experimental group		HFD + Quercetin		Feeding	4 mg/d	8 weeks	
Qi et al. (2020)	ApoE^−/−^	6 weeks	Male	40	Control group	20	chow diet	—	Feeding	—	12 weeks	①②③⑤⑥
					Experimental group	20	chow diet + ISL		Feeding	20 mg/kg/d	12 weeks	
Ding et al. (2019)	ApoE^−/−^	8 weeks	male	28	Control group	14	HFD	—	Feeding	—	12 weeks	①②③ ④⑤⑥
					Experimental group	14	HFD + Lut		Feeding	10 mg/kg/2 days	12 weeks	
Wang et al. (2018)	ApoE^−/−^	7 weeks	Male	30	Control group	15	HFD	95% or higher	Gavage	—	18 weeks	①②③ ④⑤⑥
					Experimental group	15	HFD + EGCG		Gavage	40 mg/kg/d	18 weeks	
Wang et al. (2012b)	ApoE^−/−^	8 weeks	Male	20	Control group	10	HCD	—	feeding	—	8 weeks	①②③ ④⑤⑥
					Experimental group	10	HCD + Cy-3-G		Feeding	2 g/kg	8 weeks	
Wang et al. (2021b)	ApoE^−/−^	—	Male	30	Control group	10	HFHCD	—	Feeding	—	8 weeks	①②③ ④⑤⑥
					Experimental group	10	HFHCD + Rhamnetin (low)		Feeding	100 mg/kg	8 weeks	
						10	HFHCD + Rhamnetin (high)		Feeding	200 mg/kg	8 weeks	

① aortic atherosclerotic lesion area, ② TC, ③ TG, ④ HDL-C, ⑤ LDL-C, ⑥ Mechanism of action.

**TABLE 2:** Types of flavonoids in the literature were included.

Investigator name	Year	Flavonoids	Subclasses
Wang et al. (2012b)	2012	Cyanidin - 3 - O - beta - Glucoside (Cy - 3 - G)	Anthocyanin
Sun et al. (2013)	2013	Myricitrin	Flavonl
Qi et al. (2020)	2020	Isoliquiritigenin (ISL)	Chalcone
Zeng et al. (2021)	2021	Theaflavin (TF)	Catechin
Wang et al. (2021)	2021	Rhamnetin	Flavonl
Luo et al. (2020)	2020	Quercetin	Flavonl
Auclair et al. (2009)	2009	(+)-Catechin	Catechin
Wang et al. (2018)	2018	(−)-Epigallocatechin-3-gallate (EGCG)	Catechin
Ding et al. (2019)	2019	Luteolin (Lut)	Flavone
Wu et al. (2018)	2018	Baicalin	Flavone
Wang et al. (2012a)	2012	Cyanidin - 3 - O - beta - Glucoside (Cy - 3 - G)	Anthocyanin

### 3.3 Risk assessment of literature bias

**FIGURE 3:**
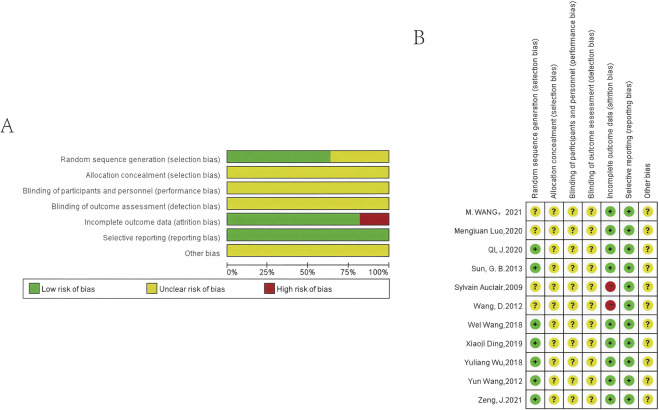
Risk of bias graph and bias summary. **(A)** Risk of bias graph, **(B)** Risk of bias summary.

Reviewers used the Cochrane Reviewers Handbook 6.1.0 for reviewers' quality evaluation of the literature (Sterne et al., 2019). A total of 11 English literature articles were included. Seven studies mentioned “random” grouping, rated as “Low risk”, and four studies did not indicate whether it was random or not, rated as “Unclear risk”. Assignment concealment was not addressed in any of the 11 studies and was categorized as “Unclear risk”. The use of blind method during drug intervention was not reported in any of the 11 studies, which was rated as “Unclear risk”. None of the 11 studies mentioned the use of blindness in the outcome measurement process, which was rated as “Unclear risk”. Nine studies reported the outcome indicators of the expected measurement, no case of early termination of the trial was found, incomplete data and biased reporting were rated as “Low risk”, and two studies reported incomplete main outcome indicators of the expected outcome as “High risk”. None of the 11 studies mentioned any other biases, which was rated as “Unclear risk”. The literature quality evaluation is shown in Figure 3.

### 3.4 Meta-analysis

#### 3.4.1 Heterogeneity test and subgroup analysis of LDL-C

**FIGURE 4:**
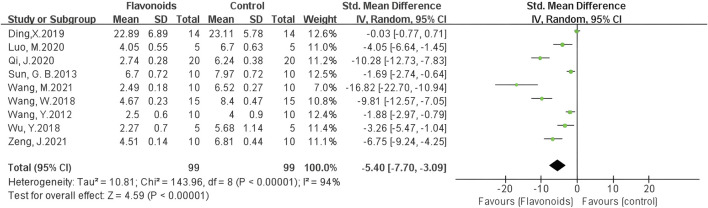
Forest map for heterogeneity test of LDL-C.

Nine of the included studies markers and included a total of 198 ApoE^−/−^ mice, including 99 in the flavonoid-administered group and 99 in the control group (Wang et al., 2012b; Sun et al., 2013; Wang et al., 2018; Wu et al., 2018; Ding et al., 2019; Luo et al., 2020; Qi et al., 2020; Wang et al., 2021; Zeng et al., 2021). According to the forest map (Figure 4), the heterogeneity test results were as follows: *Tau* ^*2*^ = 10.81, *Chi* ^*2*^ = 143.96, *df* = 8 (P < 0.00001), *I* ^*2*^ = 94%, where *I* ^*2*^ = 94% > 75%. This result indicated that there was a high degree of heterogeneity among the nine studies, so the random effects model (RE) was used to combine the effect sizes. The results of combined effect sizes showed that compared with the animal model control group, the value of LDL-C index in the flavonoid administration group was significantly reduced (SMD = −5.40, 95%CI: [−7.70, −3.09]), and *P* < 0.1 indicated that the difference was statistically significant. The above analysis indicated that flavonoids could reduce the accumulation of LDL-C in blood. The above results indicated that there was a high degree of heterogeneity among the nine studies. In order to explore the source of heterogeneity, subgroup analysis was conducted according to the route of administration, dose of administration and duration of administration of LDL-C data in the studies (Supplementary Figure S1). There was no significant change in the *P* value and *I* ^*2*^ value in the results of the three subgroup analyses, and there was still a high degree of heterogeneity, and no source of heterogeneity was found.

#### 3.4.2 Heterogeneity test and subgroup analysis of TC

**FIGURE 5:**
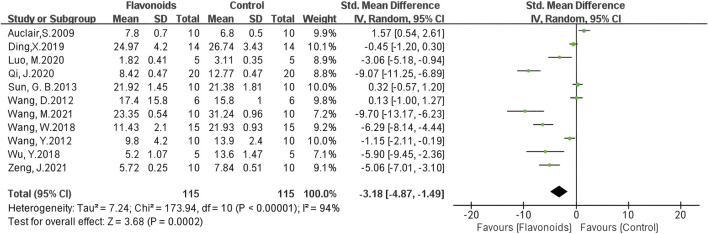
Forest map for heterogeneity test of TC.

The 11 studies (Auclair et al., 2009; Wang et al., 2012a; Wang et al., 2012b; Sun et al., 2013; Wang et al., 2018; Wu et al., 2018; Ding et al., 2019; Luo et al., 2020; Qi et al., 2020; Wang et al., 2021; Zeng et al., 2021) included reported changes in serum or plasma TC indices, including 230 ApoE^−/−^ mice, including 115 in the flavonoid administration group, and 115 in the control group. According to the forest map (Figure 5), the heterogeneity test results are as follows: *Tau* ^*2*^ = 7.24, *Chi* ^*2*^ = 173.94, *df* = 10 (P < 0.00001), *I* ^*2*^ = 94%, where *I* ^*2*^ = 94% > 75%, indicating a high degree of heterogeneity among the 11 studies, a random-effects model was employed to synthesize the effect sizes. The results of combined effect sizes showed that Compared to the control group in the animal model, the flavonoid-treated group exhibited a significant reduction in the TC index value (SMD = −3.18, 95% CI: [-4.87, -1.49]), and P < 0.1 indicated that the difference was statistically significant. The above analysis indicated that flavonoids could reduce the content of TC in blood. The results of the heterogeneity test indicated substantial heterogeneity across the 11 studies. Subgroup analysis was performed to investigate the origins of heterogeneity, examining the route of drug administration, dosage, and duration of treatment for TC data within the study (refer to Supplementary Figure S2). The subgroup analyses did not yield any significant alterations in the *P* value or *I* ^*2*^ value, indicating persistent high heterogeneity. Consequently, the analyses did not identify the source of heterogeneity.

#### 3.4.3 Heterogeneity test and subgroup analysis of TG

**FIGURE 6:**
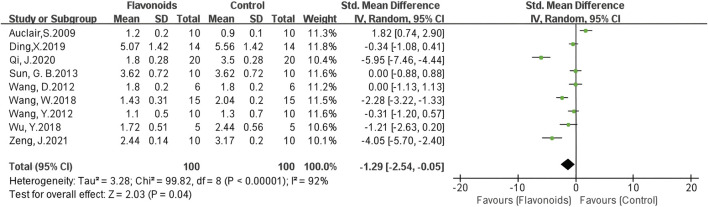
Forest map for heterogeneity test of TG.

Among the included studies, nine studies reported changes in serum or plasma TG indices, including a total of 200 ApoE^−/−^ mice, comprising 100 participants in the flavonoid group and 100 in the control group (Auclair et al., 2009; Wang et al., 2012a; Wang et al., 2012b; Sun et al., 2013; Wang et al., 2018; Wu et al., 2018; Ding et al., 2019; Qi et al., 2020; Zeng et al., 2021). According to the forest map (Figure 6), the heterogeneity test results were as follows: *Tau* ^*2*^ = 3.28, *Chi* ^*2*^ = 99.82, *df* = 8 (P < 0.00001), *I* ^*2*^ = 92%, where *I* ^*2*^ = 92% > 75%, this indicates substantial heterogeneity across the nine studies; therefore, a random-effects model (RE) was employed to aggregate the effect sizes. The results of combined effect sizes showed that compared with the animal model control group, TG index values in the flavonoid-administered group were dramatically decreased (SMD = −1.29, 95% CI: [-2.54, -0.05]), and *P* < 0.1 indicated that the difference was statistically significant. The above analysis indicated that flavonoids could reduce the content of TG in blood. The results of the heterogeneity test revealed significant variability among the nine studies. To investigate the origins of the heterogeneity, a subgroup analysis was performed according to administration route, administration dose and duration of administration of TG data in the studies (Supplementary Figure S3). There was no significant change in the *P* value and *I* ^*2*^ value in the results of the three subgroup analyses, despite thorough analysis, a high level of heterogeneity persisted, and the root cause remained unidentified.

#### 3.4.4 Heterogeneity test and subgroup analysis of HDL-C

**FIGURE 7:**
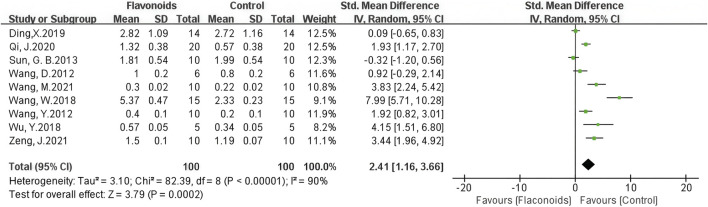
Forest map for heterogeneity test of HDL-C.

Changes in serum or plasma HDL-C markers were reported in nine studies (Wang et al., 2012a; Wang et al., 2012b; Sun et al., 2013; Wang et al., 2018; Wu et al., 2018; Ding et al., 2019; Qi et al., 2020; Wang et al., 2021; Zeng et al., 2021) in the included literature, including a total of 200 ApoE^−/−^ mice, including 100 in the flavonoid administration group and 100 in the control group. According to the forest map (Figure 7), the heterogeneity test results were as follows: *Tau* ^*2*^ = 3.10, *Chi* ^*2*^ = 82.39, *df* = 8 (P < 0.00001), *I* ^*2*^ = 90%, where *I* ^*2*^ = 90% > 75%, this suggests significant heterogeneity across the nine studies; therefore, a random-effects model was employed to amalgamate the effect sizes. The data of the combined effect sizes revealed that, in comparison to the control group in the animal model, the HDL-C index value in the flavonoid administration group was significantly increased (SMD = 2.41, 95%CI: [1.16,3.66]), and *P* < 0.1 indicated that the difference was statistically significant. The above analysis indicated that flavonoids could increase the content of HDL-C in blood. The heterogeneity test indicated substantial variability among the nine studies. Subgroup analyses were performed based on the route of administration, dosage, and duration of HDL-C administration in the research (Supplementary Figure S4) to investigate the heterogeneity’s origin. These subgroup analyses did not yield a notable shift in the *P* value or *I* ^*2*^ value, maintaining a high level of heterogeneity without identifying the underlying source.

#### 3.4.5 Sensitivity analysis

**FIGURE 8:**
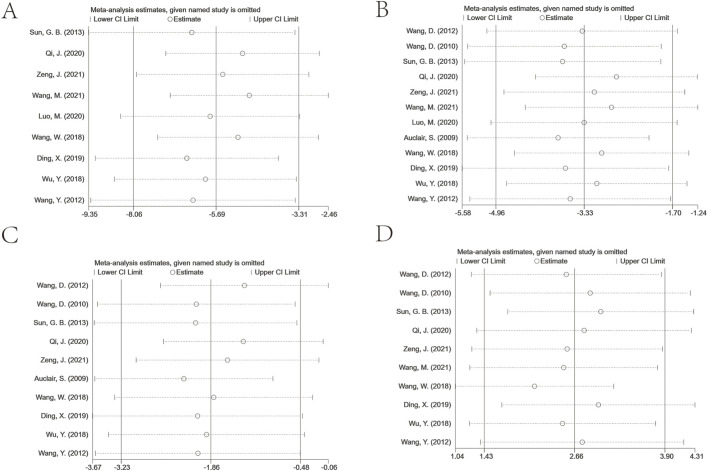
Sensitivity analysis. **(A)** LDL-C, **(B)** TC, **(C)** TG, **(D)** HDL-C.

To assess the robustness of the evaluation results, one separate study was deleted one by one, and sensitivity analysis was performed on LDL-C, TC, TG, and HDL-C indicators (Figure 8). There was no reversal of the results before and after the sensitivity analysis, suggesting that the findings for the four metrics were dependable.

#### 3.4.6 Publication bias

**FIGURE 9:**
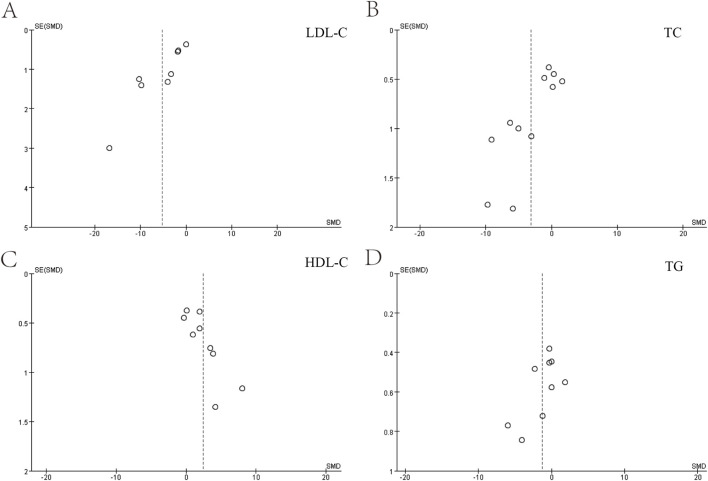
Funnel chart of lipid indicators.

**FIGURE 10:**
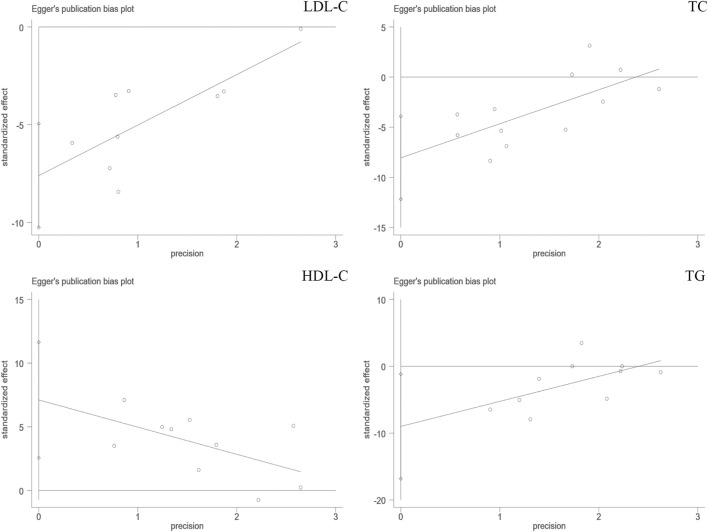
Chart of the results of Egger’s test.

**FIGURE 11:**
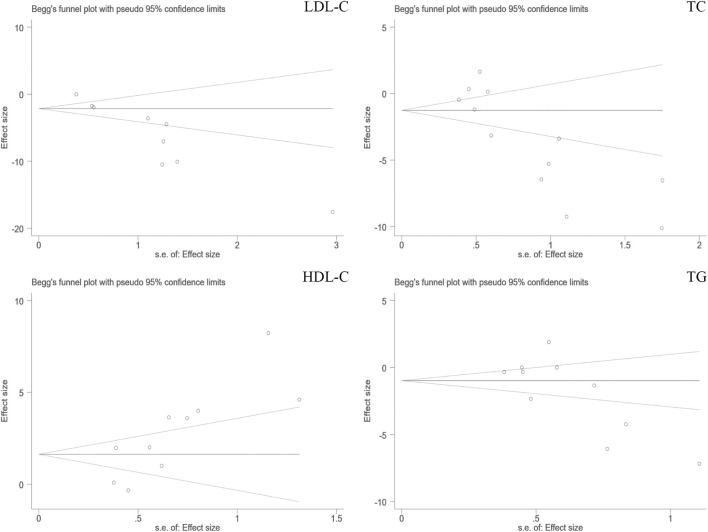
Plot of the results of Begg’s test.

The Funnel plot, Begg’s test and Egger’s test were utilized to assess the presence of the publication bias of LDL-C, TC, TG, HDL-C, and other indicators. The results are shown in Table 3, Figures 9–11. The dispersion distribution of the funnel plot of the four lipid indexes was asymmetrical, and the *P*-values in Begg’s test and Egger’s test were all less than 0.05, indicating the possible existence of publication bias.

**TABLE 3:** Results of Begg’s test and Egger’s test.

Bias test	LDL-C	TC	TG	HDL-C
Begg’s test P value	0.004	0.020	0.107	0.173
Egger’s test P value	0.011	0.006	0.030	0.143

### 3.5 Effect of flavonoids on aortic atherosclerotic plaque area

Changes in aortic atherosclerotic plaque area were reported in 11 studies. The area of aortic atherosclerotic plaque decreased in all 11 studies, suggesting that flavonoids can reduce the area of plaque, missing variance measures and non-extractable formats prevented pooling of plaque area. (Table 4).

**TABLE 4:** Changes in staining patterns and area of aortic histopathology.

Investigator name	Year	Dyeing method	Area variation
Wang et al. (2012b)	2012	Oil red O stain	The lesion area is reduced by approximately 66%
Zeng et al. (2021)	2021	Hematoxylin-eosin stain and massasone stain	Reduced lesion are
Auclair et al. (2009)	2009	Oil red O stain	The lesion area is reduced by approximately 32%
Wu et al. (2018)	2018	Hematoxylin-eosin stain	The lesion area is reduced by about 42%
Sun et al. (2013)	2013	Oil red O stain	The lesion area is reduced by about 7%
Luo et al. (2020)	2020	Oil red O stain	The lesion area was reduced by 55.49%
Qi et al. (2020)	2020	Hematoxylin-eosin stain	Reduced lesion area
Ding et al. (2019)	2019	Oil red O stain	The lesion area was reduced by about 3.84%
Wang et al. (2018)	2018	Oil red O stain	The lesion area is reduced by about 29%
Wang et al. (2012b)	2012	Oil red O stain	The lesion area is reduced by approximately 54%
Wang et al. (2021)	2021	Oil red O stain and hematoxylin-eosin stain	6.21% reduction in lesion area (HE staining) 12.71% reduction in lesion area (oil red O staining)

### 3.6 Mechanism of flavonoids on atherosclerosis

**FIGURE 12:**
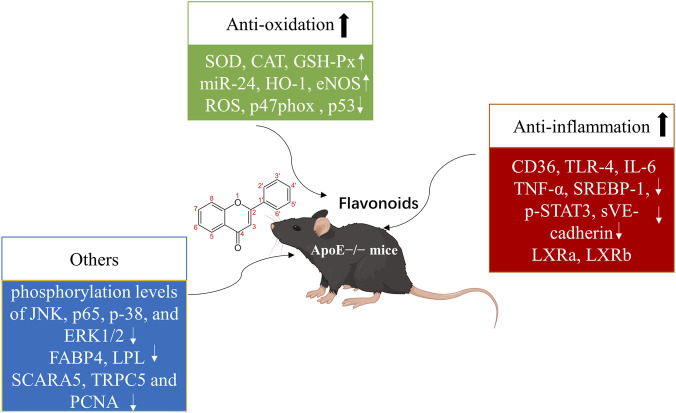
Mechanism of flavonoids on atherosclerosis in ApoE−/− mice. “↑” means upregulation, “↓” means downregulation.

As is shown is Figure 12, Supplementary Table S3, a number of mechanisms associated with flavonoids on atherosclerosis were detailed in the chosen studies. Some of the studies revealed that flavonoids scavenged reactive oxygen species (ROS), thereby mitigating oxidative stress. And some research showed that flavonoids prevented AS through inhibiting inflammation. Moreover, other studies identified different mechanism such as Nrf2/HO-1, p38/MAPK signaling pathway.

Four publications reported that the antioxidant effect of flavonoids. Zeng et al. (2021) showed that administration of theaflavin notably decreased serum lipid concentrations and the production of malondialdehyde (MDA) in mice fed a high-fat diet (HFD). Theaflavin supplementation not only significantly enhanced the activities of antioxidant enzymes such as superoxide dismutase (SOD), catalase (CAT), and glutathione peroxidase (GSH-Px), but also upregulated the expression of microRNA-24 (miR-24). Sun et al. (2013) found that myricitrin could lower ROS levels and prevent H2O2-induced endothelial injury, this effect was associated with a significant downregulation of p53 gene expression, activation of caspase-3, engagement of the MAPK pathway, and alterations in the expression patterns of anti-apoptotic genes. Luo et al. (2020) revealed that quercetin intake significantly decreased the expression of p47phox and mitigated NADPH oxidase-driven oxidative stress in the aortic tissue of HFD-fed ApoE^−/−^ mice. Concurrently, quercetin administration elevated both the expression and enzymatic activity of the antioxidant heme oxygenase-1 (HO-1). Wang et al. (2012b) reported that the anthocyanin cyanidin-3-O-β-glucoside (C3G) is capable of preventing or reversing endothelial dysfunction caused by hypercholesterolemia. It achieves this by inhibiting the buildup of cholesterol and 7-oxysterols in the aorta, which in turn leads to decreased superoxide generation. The consequent maintenance of eNOS function and the availability of nitric oxide (NO) are supported by these effects.

Three studies showed that flavonoids inhibited the inflammatory pathway in atherosclerosis. Wang et al. (2021) indicated that rhamnetin reduced the prevalence of foam cells and apoptotic cells, as well as diminished the expression levels of CD36 and TLR-4 proteins in macrophages exposed to ox-LDL. Additionally, rhamnetin lowered the expression of TLR-4 mRNA and components of the TLR-4 signaling pathway in the aortic tissues of ApoE^−/−^ mice. Wang et al. (2018) found that (−)-Epigallocatechin-3-gallate (EGCG) led to a reduction in the plasma concentrations of IL-6 and TNF-α. Concurrently, EGCG notably enhanced the expression of liver X receptor alpha (LXRα) and liver X receptor beta (LXRβ) in the liver. It also inhibited the expression of both the precursor and mature forms of sterol regulatory element-binding protein-1 (SREBP-1). Ding et al. (2019) revealed that luteolin markedly diminished the progression of atherosclerosis in ApoE^−/−^ mice fed a high-fat diet, primarily by mitigating inflammatory responses. Furthermore, *in vitro* studies showed that luteolin attenuated oxLDL-induced inflammation through the inhibition of the signal transducer and activator of transcription 3 (STAT3) pathway. Molecular modeling analyses provided additional insight, indicating that the principal mechanism of luteolin’s interaction with STAT3 is through the formation of hydrogen bonds.

One study showed duo-mechanism on oxidative stress and inflammation. Wu et al. (2018) revealed that baicalin enhanced the activity of antioxidant enzymes, including SOD, and GSH-Px, while decreasing the levels of the oxidative stress marker MDA in comparison to the AS model group, thereby confirming baicalin’s antioxidative properties. Additionally, baicalin administration reduced the elevated concentrations of pro-inflammatory cytokines, underscoring its anti-inflammatory effects in AS. Further investigation into baicalin’s mode of action revealed that it lowered the increased phosphorylation of JNK, p65, p-38, and ERK1/2 associated with AS, indicating that baicalin potentially inhibits the NF-κB and p38 MAPK signaling pathways in the context of AS.

Besides, Wang et al. (2012a) suggested that the hypocholesterolemic effect of cyanidin-3-O-glucoside may be partially attributed to its activation of the LXR-CYP7A1-bile acid excretion pathway, thereby aiding in the antiatherogenic properties of cyanidin-3-O-glucoside. Notably, cyanidin-3-O-glucoside exhibited agonist-dependent activation of LXR. Auclair et al. (2009) showed that Catechin supplementation led to a 32% reduction in the average size of atherosclerotic lesions. Catechin supplementation significantly altered the expression of 450 genes. Additionally, genes involved in energy metabolism, lipid metabolism, and lipid transport, including FABP4, LPL, and SCARA5, exhibited decreased expression, which may play a role in the atheroprotective effects attributed to catechin. Qi et al. (2020) observed that isoliquiritigenin could mitigate atherosclerotic lesions, lower serum lipid levels, and suppress TRPC5 expression in ApoE^−/−^ mice. In cell culture experiments, isoliquiritigenin inhibited the proliferation of vascular smooth muscle cells (VSMCs) stimulated by Ang II and dose-dependently reduced Ang II-induced expressions of TRPC5 and PCNA.

## 4 Discussion

In recent research, a range of flavonoids have been explored for their potential in treating atherosclerosis (Auclair et al., 2009; Ding et al., 2019; Ciumărnean et al., 2020; Gouveia et al., 2022). The central concern lies in the consistency and reliability of the findings from these research studies.

Our thorough review of 11 studies focusing on the efficacy of flavonoids in ApoE^−/−^ mouse atherosclerosis, selected from multiple databases based on predefined criteria, revealed compelling evidence supporting the effectiveness of flavonoids in treating atherosclerosis. In contrast to previous work such as Liao et al. , this study uniquely focuses on ApoE^−/−^ mousemodels and integrates lipid outcomes, aortic plaque measures, and mechanistic pathways. Given the small number of male-only studies, findings should be interpreted as hypothesis-generating. Based on the findings of the aforementioned meta-analysis, flavonoids demonstrate the ability to decrease the levels of LDL-C, TC, and TG in the bloodstream compared to ApoE^−/−^ mice. Research has highlighted the role of subcutaneous retention and oxidation of LDL within arteries in the formation and instability of atherosclerotic plaques (Tian et al., 2020). Flavonoids found in natural medicines exhibit promise in treating atherosclerosis by inhibiting LDL-C oxidation or directly interacting with lipoproteins in LDL to modulate cellular antioxidant status (Zhang et al., 2021). Following flavonoid administration, a significant reduction in LDL-C levels in the blood and varying degrees of reduction in aortic atherosclerotic lesion areas were observed, indicating a therapeutic effect on atherosclerosis (Liao et al., 2023). Among the included studies, one dataset yielded an SMD of −0.03, indicating no meaningful LDL-C–lowering effect and representing a clear outlier relative to the other findings. To evaluate its influence, we performed an additional sensitivity analysis by excluding this study. The pooled effect size remained significant and directionally consistent, demonstrating that the overall LDL-C result is not driven by this single outlier. Different types of flavonoids exhibit specific structure-activity relationships, effective concentrations, and potential synergistic or antagonistic effects with other natural antioxidants or compounds. Across all four lipid outcomes, none of the subgroup analyses produced a meaningful reduction in heterogeneity (all I^2^ values remained ≥75%). Similarly, the direction and magnitude of effect estimates within subgroups were generally consistent with the overall pooled estimates. These findings indicate that the examined moderators do not sufficiently explain the variability, and other unmeasured factors—such as flavonoid purity, differences in diet composition, or variation in plaque quantification techniques—may contribute to the observed heterogeneity. Detailed subgroup results are provided in Supplementary Figures S1–S4. Despite the presence of significant heterogeneity among the studies, subgroup analysis was unable to pinpoint the source of this variation based on factors such as administration route, dosage, and duration. The lack of significant changes in P-values and I^2^ values in the subgroup analysis indicates the absence of identifiable sources of heterogeneity. Moreover, the asymmetrical distribution of the funnel plot and P-values below 0.05 in Begg’s and Egger’s tests suggest potential publication bias. Our risk-of-bias assessment revealed variability in methodological rigor among the included studies. Although several studies reported random allocation, none provided sufficient detail regarding allocation concealment, and blinding of investigators or outcome assessors was rarely described. These omissions increase the risk of selection and detection bias and may influence outcome measurements such as lesion area quantification or lipid profile assessment. Additionally, two studies had incomplete outcome reporting, which raises concerns about selective reporting bias. These methodological weaknesses likely contribute to the heterogeneity observed in our analyses and reduce the overall certainty of the evidence. Strengthening reporting standards in future animal studies—including adherence to ARRIVE 2.0 guidelines—would enhance comparability and reliability.

We meticulously presented the pharmacological mechanisms of each study and assessed methodological quality, offering valuable insights for guiding future trials and avoiding unnecessary duplication. Certain studies have demonstrated that flavonoids are capable of scavenging ROS to mitigate oxidative stress. Additionally, research has indicated that flavonoids can hinder atherosclerosis (AS) by suppressing inflammation. Furthermore, other studies have identified various mechanisms involved, including the Nrf2/HO-1 pathway and the p38/MAPK signaling pathway. However, this study has limitations. The inclusion of only 10 flavonoids and five flavonoid subtypes may not encompass the full spectrum of flavonoids. Additionally, two studies were deemed high risk due to missing LDL-C data, potentially introducing bias. Inadequate blinding in intervention processes and incomplete or uncertain reporting of evaluation items further underscore the need for cautious interpretation. Moreover, the limited availability of data on inflammatory factors restricted the scope of analysis. While subgroup analysis did not reveal heterogeneity sources for lipid indicators, factors such as flavonoid type and modeling methods could still contribute to variability. The exclusion of non-English literature and the small sample sizes of outcome indicators may lead to potentially skewed results.

## 5 Conclusion

Overall, we conducted a systematic review of 11 studies assessing the Anti-atherosclerotic effect of flavonoids in ApoE^−/−^ mice models, which were meticulously selected from three databases. The analysis indicates that flavonoids possess a certain therapeutic efficacy against atherosclerosis, with varying effects among different drugs on reducing aortic atherosclerotic lesions. Flavonoids exhibit the capacity to lower LDL-C, TC, and TG levels in the blood while increasing HDL-C levels. However, further high-quality clinical evidence derived from randomized controlled trials within atherosclerosis populations and diverse drug-related studies is essential to establish flavonoids as a reliable treatment option. Although ApoE^−/−^ mice represent a well-established model for studying lipid dysregulation and plaque formation, they do not fully mimic human atherosclerosis. Differences in lipid metabolism, inflammatory responses, and especially flavonoid absorption and biotransformation limit the ability to generalize these findings to humans. The dosages used in animal studies often exceed those achievable through dietary intake, and flavonoid pharmacokinetics vary substantially between species. For these reasons, our conclusions regarding clinical relevance must be interpreted with caution. Well-designed human trials are needed to determine whether the benefits observed in mice translate to meaningful cardiovascular outcomes in patients.

## Data Availability

The original contributions presented in the study are included in the article/Supplementary Material, further inquiries can be directed to the corresponding authors.
