# Supplementary figures and images for: Effects of flavonoids on atherosclerosis in ApoE−/− mice: a systematic review and meta-analysis
Source: Front Pharmacol. 2026 May 29;17:1816659. doi: 10.3389/fphar.2026.1816659 (PMC13260573; doi:10.3389/fphar.2026.1816659)

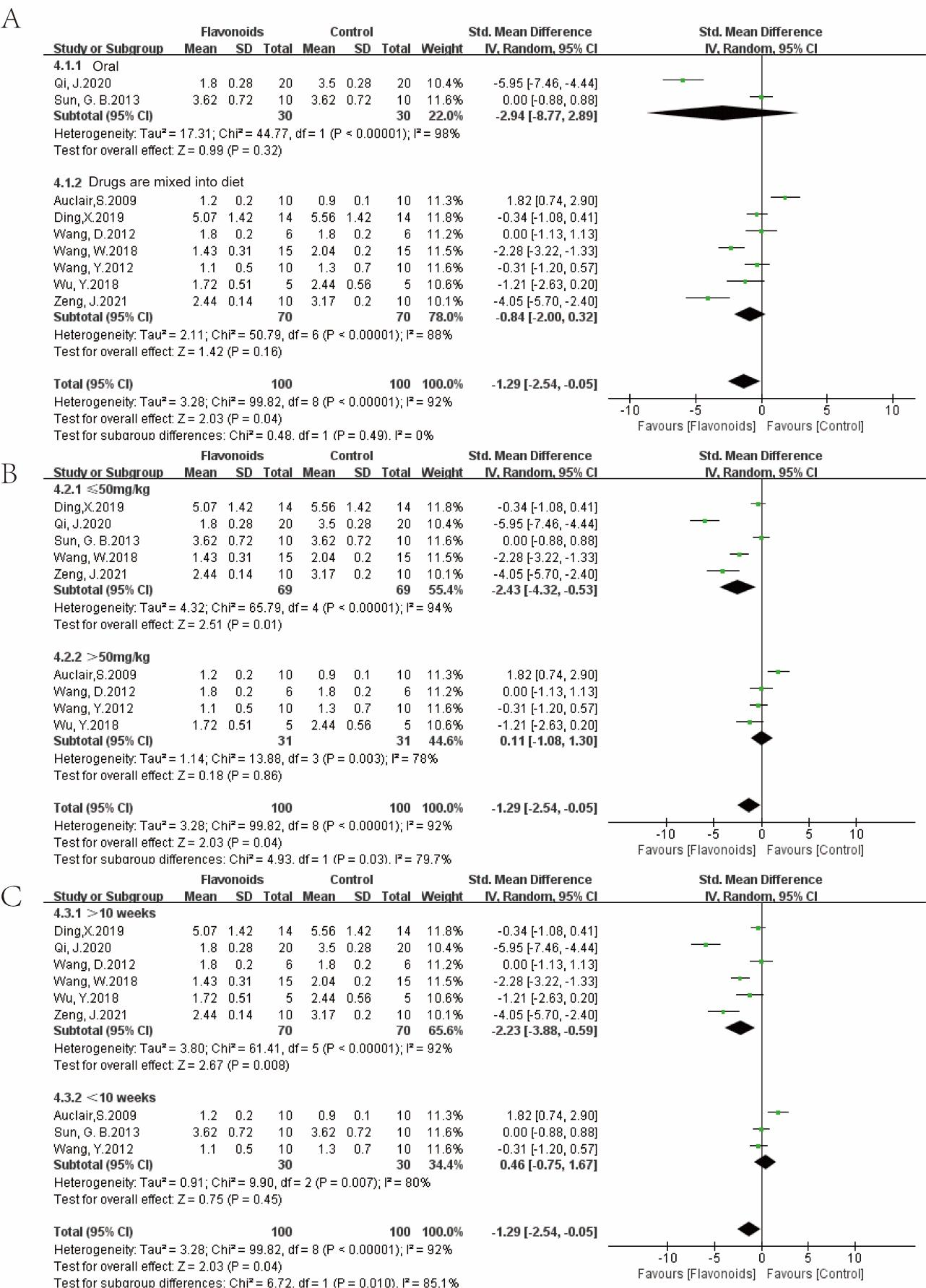

Supplement: Supplementary file 1 [file Image3.tiff]

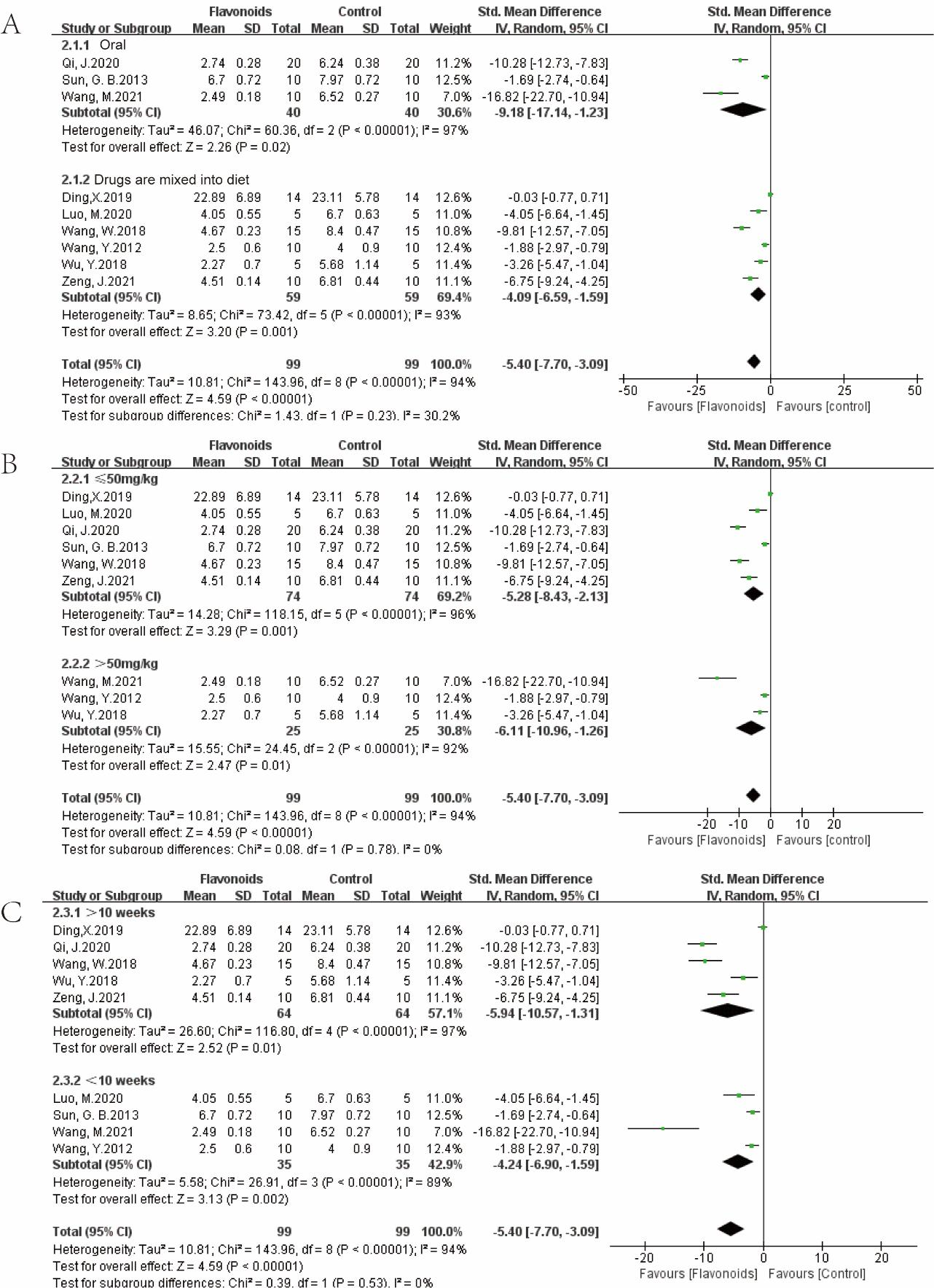

Supplement: Supplementary file 2 [file Image1.tiff]

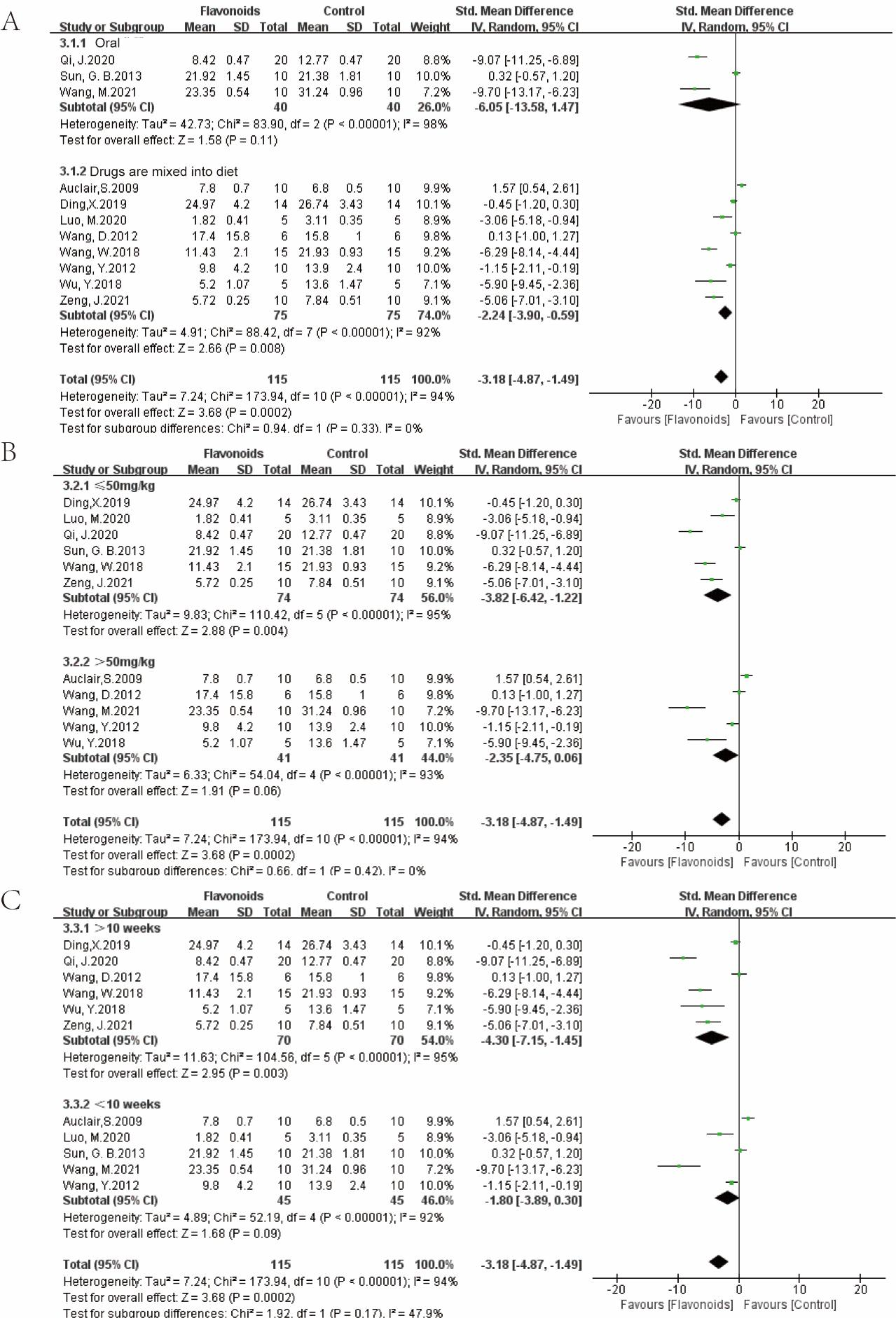

Supplement: Supplementary file 4 [file Image2.tiff]

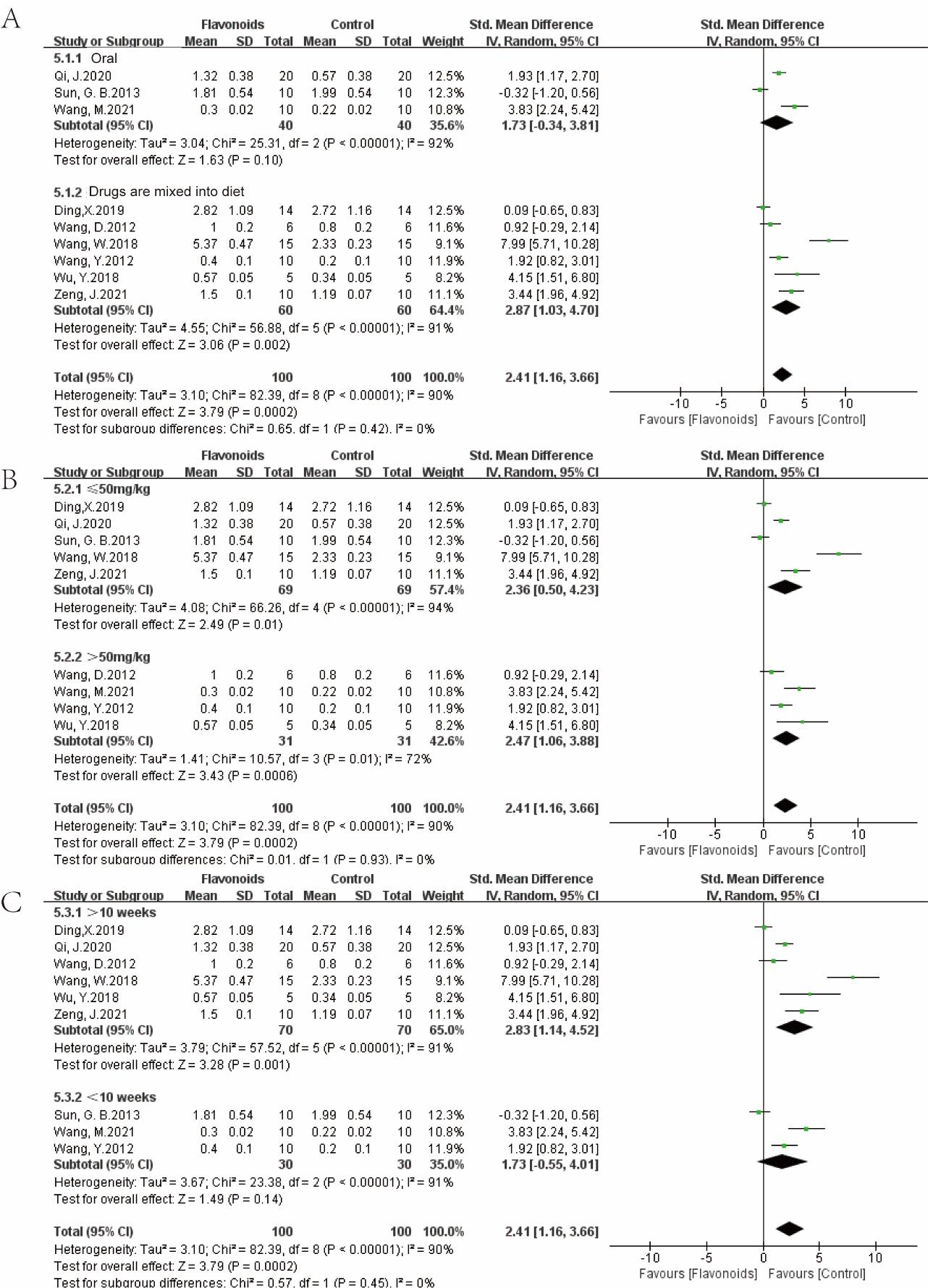

Supplement: Supplementary file 5 [file Image4.tiff]
