# Supplementary material for: Effects of flavonoids on atherosclerosis in ApoE−/− mice: a systematic review and meta-analysis
Source: Front Pharmacol. 2026 May 29;17:1816659. doi: 10.3389/fphar.2026.1816659 (PMC13260573; doi:10.3389/fphar.2026.1816659)
Supplement: Supplementary file 3 [file Supplementaryfile1.docx]

Supplementary Material

**Supplemental Tables and Figures**

**Supplemental Table1.** Summary of subject words and free words

|  | **Subject Words** | **Free Words** |
| --- | --- | --- |
| **Flavonoids** | Flavonoids | 2-Phenyl-Chromenes  2 Phenyl Chromenes  2-Phenyl-Benzopyran  2 Phenyl Benzopyran  2-Phenyl-Benzopyrans  2 Phenyl Benzopyrans  2-Phenyl-Chromene  2 Phenyl Chromene  Flavonoids  Bioflavonoids  Bioflavonoids |
| **Atherosclerosis** | Atherosclerosis | Atheroscleroses  Atherogenesis  Atherogeneses |

**Supplemental Table2.** Search strategies and search queries of each database

| **Datebase** | **Number** | **Search terms** |
| --- | --- | --- |
| **PubMed** | # 1 | (flavonoids[Title/Abstract]) OR (2-Phenyl-Chromenes[Title/Abstract])) OR (2 Phenyl Chromenes[Title/Abstract])) OR (2-Phenyl-Benzopyran[Title/Abstract])) OR (2 Phenyl Benzopyran[Title/Abstract])) OR (2-Phenyl-Benzopyrans[Title/Abstract])) OR (2 Phenyl Benzopyrans[Title/Abstract])) OR (2-Phenyl-Chromene[Title/Abstract])) OR (2 Phenyl Chromene[Title/Abstract])) OR (Flavonoids[Title/Abstract])) OR (Bioflavonoids[Title/Abstract])) OR (Bioflavonoids[Title/Abstract]) |
|  | # 2 | (((atherosclerosis[Title/Abstract]) OR (Atheroscleroses[Title/Abstract])) OR (Atherogenesis[Title/Abstract])) OR (Atherogeneses[Title/Abstract]) |
|  | # 3 | #1 AND #2 |
| **Web of Science** | # 1 | TS=(flavonoids OR 2-Phenyl-Chromenes OR 2 Phenyl Chromenes OR 2-Phenyl-Benzopyran OR 2 Phenyl Benzopyran OR 2-Phenyl-Benzopyrans OR 2 Phenyl Benzopyrans OR 2-Phenyl-Chromene OR 2 Phenyl Chromene OR Flavonoids OR Bioflavonoids OR Bioflavonoids) AND TS=(atherosclerosis OR Atheroscleroses OR Atherogenesis OR Atherogeneses) |
| **EMBASE** | # 1 | 'flavonoids'/exp OR flavonoids OR '2-phenyl-chromenes' OR '2 phenyl chromenes' OR '2-phenyl-benzopyran' OR '2 phenyl benzopyran' OR '2-phenyl-benzopyrans' OR '2 phenyl benzopyrans' OR '2-phenyl-chromene' OR '2 phenyl chromene' OR 'flavonoids' OR 'bioflavonoids' OR 'bioflavonoids' |
|  | # 2 | 'atherosclerosis'/exp OR atherosclerosis OR 'atheroscleroses' OR 'atherogenesis' OR 'atherogeneses' |
|  | # 3 | #1 AND #2 |
|  | # 4 | #3 AND ('animal experiment'/de OR 'animal model'/de) |
| **Cochrane Library** | # 1 | MeSH descriptor: [Flavonoids] explode all trees |
|  | # 2 | (2-Phenyl-Chromenes OR 2 Phenyl Chromenes OR 2-Phenyl-Benzopyran OR 2 Phenyl Benzopyran OR 2-Phenyl-Benzopyrans OR 2 Phenyl Benzopyrans OR 2-Phenyl-Chromene OR 2 Phenyl Chromene OR Flavonoids OR Bioflavonoids OR Bioflavonoids) |
|  | # 3 | #1 OR #2 |
|  | # 4 | MeSH descriptor: [Atherosclerosis] explode all trees |
|  | # 5 | (Atheroscleroses OR Atherogenesis OR Atherogeneses) |
|  | # 6 | #4 OR #5 |
|  | # 7 | #3 AND #6 |

**Supplemental Table3.** Mechanism of action of flavonoids on atherosclerosis

| **Investigator name** | **Year** | **Mechanism of action** |
| --- | --- | --- |
| Wang, D. | 2012 | The cholesterol-lowering effects of Cy-3-G may be through activation of the underlying LXRA-CYP7A1-bile acid excretion pathway in the body, and Cy-3-G can activate LXRa in an agonist-dependent manner. |
| Sun, G. B. | 2013 | Myricetin protects EC (endothelial cells) from ROS (reactive oxygen species) -induced apoptosis and prevents AS formation. |
| Qi, J. | 2020 | ISL improves atherosclerosis and inhibits the proliferation of primary VSMC in mouse models. |
| Zeng, J. | 2021 | Theaflavins may achieve anti-atherosclerotic effects by activating the Mir-24-mediated Nrf2/HO-1 signaling pathway. |
| Wang, M. | 2021 | Rhamnetin therapy inhibits inflammation and pro-atherogenic pathways in ApoE^-/-^ mice by modulating the TLR-4/IkBa/NF-kBp65 pathway. |
| Luo, M. | 2020 | Dietary quercetin significantly inhibits the formation of vascular ROS and endothelial dysfunction in atherosclerotic animals; These beneficial effects of dietary quercetin are associated with the regulation of aortic NADPH oxidase and HO-1. |
| Auclair, S. | 2009 | Catechins regulate the expression of many genes involved in signal transduction, such as key regulators of ERK and p38, MAPK signaling pathways. |
| Wang, W. | 2018 | EGCG modulates high-fat mediated liver TTC39B expression, and EGCG administration mitigated atherosclerotic plaque formation and systemic chronic inflammatory processes in atherosclerotic mice. |
| Ding, X. | 2019 | Luteolin inhibits oxLDL-induced inflammation by inhibiting STAT3 activation. |
| Wu, Y. | 2018 | Baicalin alleviates atherosclerosis by relieving oxidative stress and inflammatory responses by inactivating NF-κB and p38 MAPK signaling pathways. |
| Wang, Y. | 2012 | Cy-3-G reduces cholesterol and 7-oxysterol levels through the ABCG1 pathway, thereby improving endothelial dysfunction and atherosclerosis caused by hypercholesterolemia, thereby reducing superoxide production and enhancing eNOS activity and no bioavailability. |

**Supplemental Table4. Lipid unit conversion**

| **Investigator name** | **Lipid outcomes reported** | **Original units in article** | **Standardized units used for pooling** | **Conversion method** |
| --- | --- | --- | --- | --- |
| Wang, D. (Wang et al., 2012b) | TC | mmol/L | mmol/L |  |
|  | TG | mmol/L | mmol/L |  |
|  | HDL-C | mmol/L | mmol/L |  |
| Zeng, J. (Zeng et al., 2021) | LDL-C | mmol/L | mmol/L |  |
|  | TC | mmol/L | mmol/L |  |
|  | TG | mmol/L | mmol/L |  |
|  | HDL-C | mmol/L | mmol/L |  |
| Auclair, S. (Auclair et al., 2009) | TC | mmol/L | mmol/L |  |
|  | TG | mmol/L | mmol/L |  |
| Wu, Y. (Wu et al., 2018b) | LDL-C | mmol/L | mmol/L |  |
|  | TC | mmol/L | mmol/L |  |
|  | TG | mmol/L | mmol/L |  |
|  | HDL-C | mmol/L | mmol/L |  |
| Sun, G. B. (Sun et al., 2013) | LDL-C | mmol/L | mmol/L |  |
|  | TC | mmol/L | mmol/L |  |
|  | TG | mmol/L | mmol/L |  |
|  | HDL-C | mmol/L | mmol/L |  |
| Luo, M. (Luo et al., 2020) | LDL-C | mM | mmol/L | 1mM=1mmol/L |
|  | TG | mM | mmol/L | \| 1mM=1mmol/L \| \| --- \| |
| Qi, J. (Qi et al., 2020) | LDL-C | mmol/L | mmol/L |  |
|  | TC | mmol/L | mmol/L |  |
|  | TG | mmol/L | mmol/L |  |
|  | HDL-C | mmol/L | mmol/L |  |
| Ding, X.(Ding et al., 2019) | LDL-C | mmol/L | mmol/L |  |
|  | TC | mmol/L | mmol/L |  |
|  | TG | mmol/L | mmol/L |  |
|  | HDL-C | mmol/L | mmol/L |  |
| Wang, W. (Wang et al., 2018) | LDL-C | mg/dL | mmol/L | 1 mg/dL = 0.02586 mmol/L |
|  | TC | mg/dL | mmol/L | 1 mg/dL = 0.02586 mmol/L |
|  | TG | mg/dL | mmol/L | 1 mg/dL = 0.011299 mmol/L |
|  | HDL-C | mg/dL | mmol/L | 1 mg/dL = 0.02586 mmol/L |
| Wang, Y. (Wang et al., 2012d) | LDL-C | mmol/L | mmol/L |  |
|  | TC | mmol/L | mmol/L |  |
|  | TG | mmol/L | mmol/L |  |
|  | HDL-C | mmol/L | mmol/L |  |
| Wang, M. (Wang et al., 2021a) | LDL-C | mg/dL | mmol/L | 1 mg/dL = 0.02586 mmol/L |
|  | TC | mg/dL | mmol/L | 1 mg/dL = 0.02586 mmol/L |
|  | HDL-C | mg/dL | mmol/L | 1 mg/dL = 0.02586 mmol/L |

**Supplementary Figure 1.** Forest map for subgroup analysis of LDL-C. (A) Forest map of LDL-C subgroup analysis (route of administration), (B) Forest map of LDL-C subgroup analysis (Route of administration), (C) Forest map of LDL-C subgroup analysis (duration of administration).

**Supplementary Figure 2.** Forest map for subgroup analysis of TC. (A) Forest map of TC subgroup analysis (route of administration), (B) Forest map of TC subgroup analysis (Route of administration), (C) Forest map of TC subgroup analysis (duration of administration).

**Supplementary Figure 3.** Forest map for subgroup analysis of TG. (A) Forest map of TG subgroup analysis (route of administration), (B) Forest map of TG subgroup analysis (Route of administration), (C) Forest map of TG subgroup analysis (duration of administration).

**Supplementary Figure 4.** Forest map for subgroup analysis of HDL-C. (A) Forest map of HDL-C subgroup analysis (route of administration), (B) Forest map of HDL-C subgroup analysis (Route of administration), (C) Forest map of HDL-C subgroup analysis (duration of administration).
